# Characterization of electroforming-free titanium dioxide memristors

**DOI:** 10.3762/bjnano.4.55

**Published:** 2013-08-07

**Authors:** John Paul Strachan, J Joshua Yang, L A Montoro, C A Ospina, A J Ramirez, A L D Kilcoyne, Gilberto Medeiros-Ribeiro, R Stanley Williams

**Affiliations:** 1 nanoElectronics Research Group, HP Labs, 1501 Page Mill Rd, Palo Alto, CA 94304, USA; 2 Brazilian Nanotechnology National Laboratory, CP 6192, Campinas, SP 13083-970, Brazil; 3 Universidade Federal de Minas Gerais, 31270-901, Belo Horizonte, MG, Brazil; 4 Advanced Light Source, Lawrence Berkeley National Laboratory, Berkeley, CA 94720, USA

**Keywords:** electron microscopy, memristor, resistance switching, transition-metal oxide, X-ray spectroscopy

## Abstract

Metal–insulator–metal (MIM) structures based on titanium dioxide have demonstrated reversible and non-volatile resistance-switching behavior and have been identified with the concept of the memristor. Microphysical studies suggest that the development of sub-oxide phases in the material drives the resistance changes. The creation of these phases, however, has a number of negative effects such as requiring an elevated voltage, increasing the device-to-device variability, damaging the electrodes due to oxygen evolution, and ultimately limiting the device lifetime. In this work we show that the deliberate inclusion of a sub-oxide layer in the MIM structure maintains the favorable switching properties of the device, while eliminating many of the negative effects. Electrical and microphysical characterization of the resulting structures was performed, utilizing X-ray and electron spectroscopy and microscopy. In contrast to structures which are not engineered with a sub-oxide layer, we observed dramatically reduced microphysical changes after electrical operation.

## Introduction

A memristor is a passive electronic element that displays a pinched hysteresis loop in its current–voltage characteristic, including the resistance switching that is seen in metal–insulator–metal (MIM) devices, often called resistive random access memory (RRAM or ReRAM). The memristor concept was developed by Chua [1–2], and much later associated with the behaviors seen in a range of nanoscale devices [3–4]. In turn, the research effort to understand and develop oxide-based resistance switching devices for non-volatile memory applications has an even longer history [5–15] and remains active today. An important topic that has remained a challenge is understanding the microphysical changes [16–17] during electrical operation (forming and switching). Fortunately, physical characterization efforts with the required spatial resolution and material sensitivity are beginning to shed light on the material changes that take place in material systems such as the titanates, NiO, and HfO_2_ [18–24].

Recently, physical characterization of Pt/TiO_2_/Pt unipolar [25] and bipolar [26] resistance-switching devices by TEM and X-ray absorption revealed that the switching involves the creation of localized channels of Ti_4_O_7_ within the matrix material. The Ti_4_O_7_ channel, which can be considered an ordered array of oxygen vacancies in a TiO_2_ rutile phase, is a thermodynamically stable and metallic (at room temperature) Magnéli phase. In the unipolar switching mode [25], this conductive channel is created and destroyed during ON and OFF switching, respectively, and spans the device structure between the top and bottom electrodes. In bipolar devices [26], this conductive phase is developed during the electroforming step [27–28] and ON and OFF switching does not appear to substantially modify this phase, but instead seems to modulate a thinner insulating barrier between the channel and a metal contact [29–30]. Indeed, the generation of the Ti_4_O_7_ Magnéli phase from TiO_2_ can require significant power dissipation, lead to material damage to the device as oxygen gas evolves, and, in the case of bipolar devices, require an initial irreversible “electroforming” step to enable the subsequent reversible resistance switching to be possible. This electroforming step requires a large voltage and leads to variance from device to device. In addition, the high probability of electroforming failure results in a low switchable device yield [27]. Thus, it has been highly desirable from a technological standpoint to eliminate this step. Scientifically, it is also interesting to learn whether the formation of both a sub-oxide and an ordered phase, as seen in the Magnéli phase observations, are necessary preconditions to allow resistance switching in the titanium oxide system. The above studies point strongly to the benefits of deliberate oxide-layer engineering in order to fabricate devices that are able to switch reversibly without the need to form a conductive phase through the bulk first. Earlier studies [27] by our team indeed demonstrated that using a switching layer which includes a highly non-stoichiometric TiO_2−_*_x_* film in addition to a thin stoichiometric TiO_2_ layer can eliminate the need for an electroforming step while maintaining device performance. In the present work, we investigate the detailed material changes involved in such structures with X-ray spectromicroscopy and transmission electron microscopy (TEM), both techniques previously employed in observing the formation of a bulk conducting (Magnéli) phase. These techniques are applied to both the standard (electroformed) and non-stoichiometric (forming-free) devices in order to compare the material changes in each of them.

## Sample preparation and resistance switching

MIM crossbar devices were fabricated on a silicon/silicon-nitride substrate. In some areas, the underlying Si was etched to form free-standing membranes allowing transmission characterization by X-ray absorption spectromicroscopy and electron microscopy. The bottom and top electrodes consisted of Cr(5 nm)/Pt(15 nm) and Pt(30 nm), respectively, patterned perpendicular to each other by photolithography. In between these electrodes, a blanket switching layer was deposited with two different compositions. For standard devices, an amorphous single layer TiO_2_ (30 nm) was sputter-deposited from a titania source. For electroforming-free devices (described below), a bilayer was used consisting of TiO_2−_*_x_* (30 nm) and TiO_2_ (5 nm), where the thicker oxygen deficient layer was sputter deposited from a Ti_4_O_7_ Magnéli phase target and the thinner stoichiometric layer was again deposited from a stoichiometric titania target. Device junction areas studied included 1.5 × 1.5 μm^2^ and 3 × 3 μm^2^. The device area is defined by the overlap of the bottom and top electrode. X-ray diffraction (XRD) and selected area electron diffraction (SAED) showed that the stoichiometric TiO_2_ film is predominantly amorphous with some small (<10 nm) anatase grains, while the bilayer film is amorphous with no observed structural ordering.

**Figure 1:**
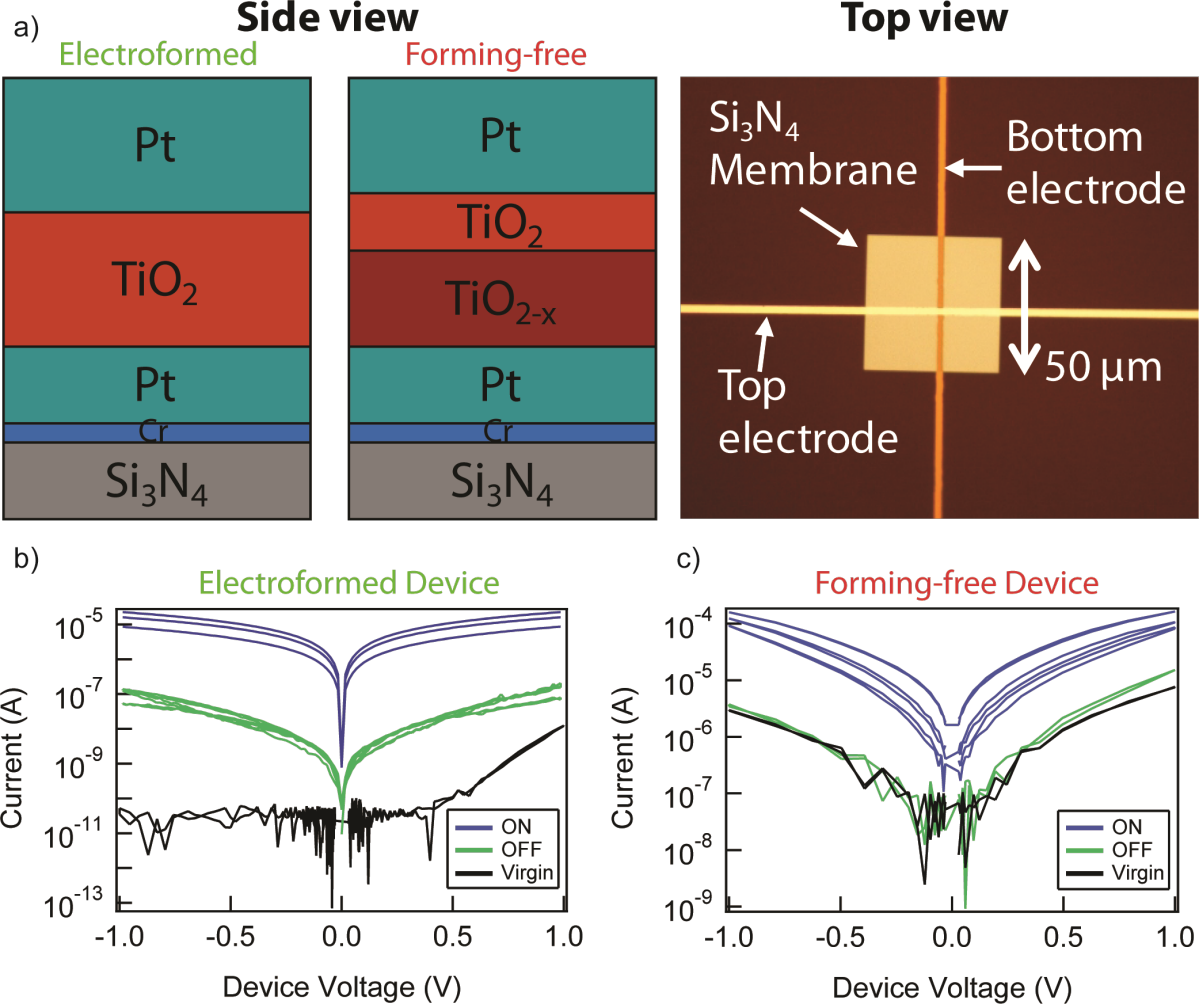
(a) Device schematics from side and top views for the electroformed (left panel) and forming-free (center panel) structures. A top view (right panel) shows the free-standing membrane allowing X-ray transmission measurements. Electrical characterization of (b) normal Pt/TiO_2_/Pt and (c) double layer Pt/TiO_2−_*_x_*/TiO_2_ device, showing the Virgin, OFF (high resistance), and ON (low resistance) states. The normal device exhibits an irreversible forming step, while the double layer structure is able to regain the Virgin state when turned OFF.

Electrical measurements of both types of devices are shown in Figure 1. Both showed reversible bipolar resistance switching. The standard device required an initial electroforming step which creates a channel of reduced titanium oxide [26], increasing the conductivity and allowing subsequent bipolar resistance switching to take place. As shown in Figure 1a, the initial high-resistance state (“Virgin”) can never be attained and thus the electroforming step is irreversible. In contrast, the bilayer device, Figure 1b, did not require such an electroforming step and subsequent bipolar resistance switching showed that the initial conductive state of the device (“Virgin”) is nearly equivalent to the subsequent OFF state. Thus, this bilayer device has three distinct properties: 1) Elimination of the higher power electroforming step, 2) the first ON switching is identical to subsequent ON switching steps, and 3) the initial Virgin state is nearly equivalent to the subsequent OFF state, thus showing that the initial state of the device can be regained, even after going to the high conductance state (ON). These three properties can be used to define a so called “forming-free” device.

## Results and Discussion

### Device characterization by X-ray spectromicroscopy

To probe any switching-induced material changes in the devices, characterization was performed using scanning transmission X-ray microscopy (STXM) at the Advanced Light Source in the Lawrence Berkeley National Laboratory. STXM allows spatially-resolved X-ray absorption spectroscopy (XAS) to be performed on a sample and is well-suited for chemical and structural characterization of the thin oxide layer within a memristor device in a non-destructive manner. High-brightness, monochromatic X-rays from a bending magnet are focused using a zone plate lens to approximately 35 nm diameter, with energy resolution of better than 100 meV [31]. In our study, the X-ray energy was swept through the Ti L-edge and for each energy point, the X-ray focus was spatially scanned to acquire an image of the device junction area. Localized spectra were extracted by processing this dataset and integrating pixels within separate regions of the device and thereby deriving the XAS intensity versus energy. This technique has been used for previous studies of the single layer TiO_2_ memristor devices and can identify the presence of spatially-localized channels of oxygen vacancy rich Ti–O phases through analysis of the near-edge X-ray absorption fine structure (NEXAFS) [26].

**Figure 2:**
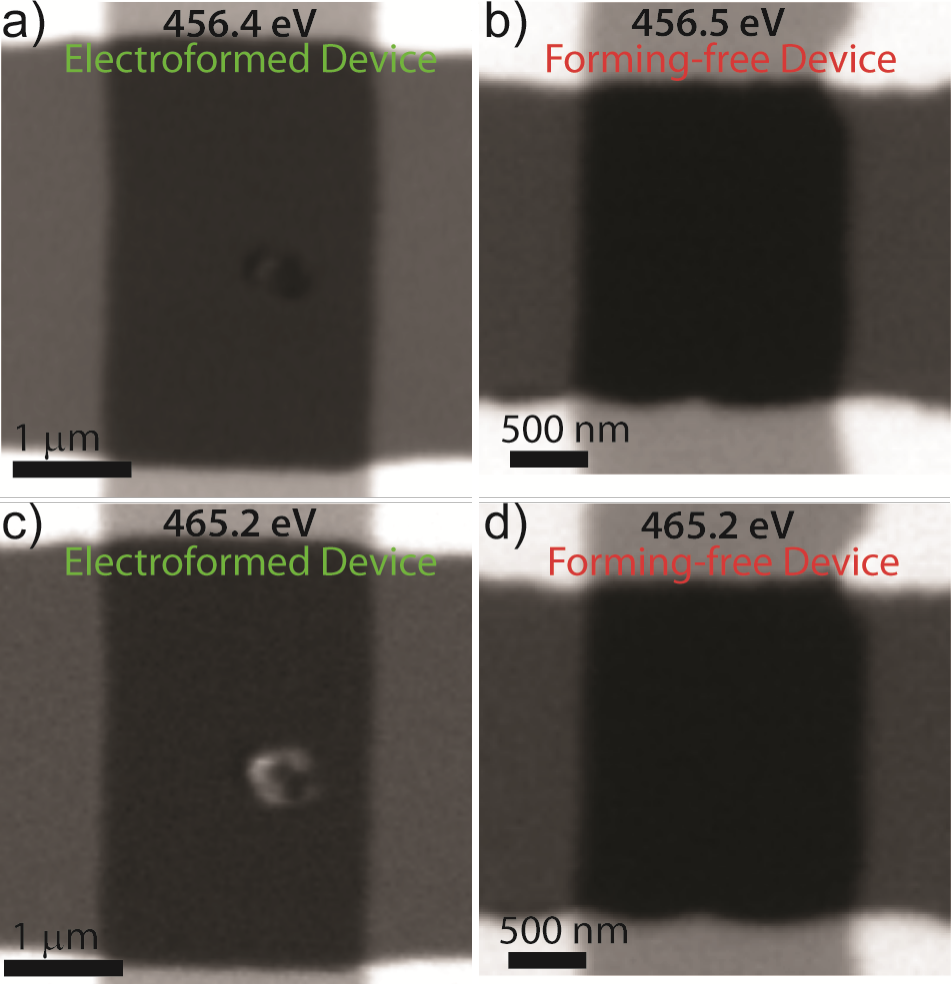
Comparison of scanning transmission X-ray micrographs for electroformed and forming-free devices. Contrast was derived from spatially-resolved X-ray absorption using incident monochromatic X-rays at the indicated energy which is before (456.4/456.5 eV) and within (465.2 eV) the main Ti L_3_ edge. For the electroformed device, (a) and (c), a strong contrast was observed within the junction which reversed at the different X-ray energies, and corresponds to the formation of a sub-oxide phase. No such contrast was observed within the junction of the forming-free devices, (b) and (d). More detailed X-ray absorption spectroscopy for these junction regions are shown below in Figure 3.

After electrical operation of the memristor devices, STXM measurements were made. Figure 2 compares post-switching X-ray absorption images of a normal electroformed device and a forming-free bilayer device. All images were taken at X-ray energies within the Ti L_2,3_ absorption edge (455–475 eV) which is sensitive to chemical and structural changes [32–34] of the titanium. An additional advantage is that by tuning to a Ti-absorption resonance, contributions from the electrodes (Pt) and other non-Ti elements in the stack are eliminated. In the images of Figure 2, regions with higher absorption are shown with darker contrast, and background absorbing materials such as the Pt electrodes with a higher elemental mass than the switching layer show up prominently. Nonetheless, in both images of the electroformed device (Figure 2a and Figure 2c), additional contrast was observed within the junction. This contrast is indicative of significant material changes within the device. The forming-free bilayer device, on the other hand, showed no spatial contrast anywhere within or near the device junction. In total, three forming-free devices were studied in STXM after resistance switching and no spatial features indicating material changes were observed.

**Figure 3:**
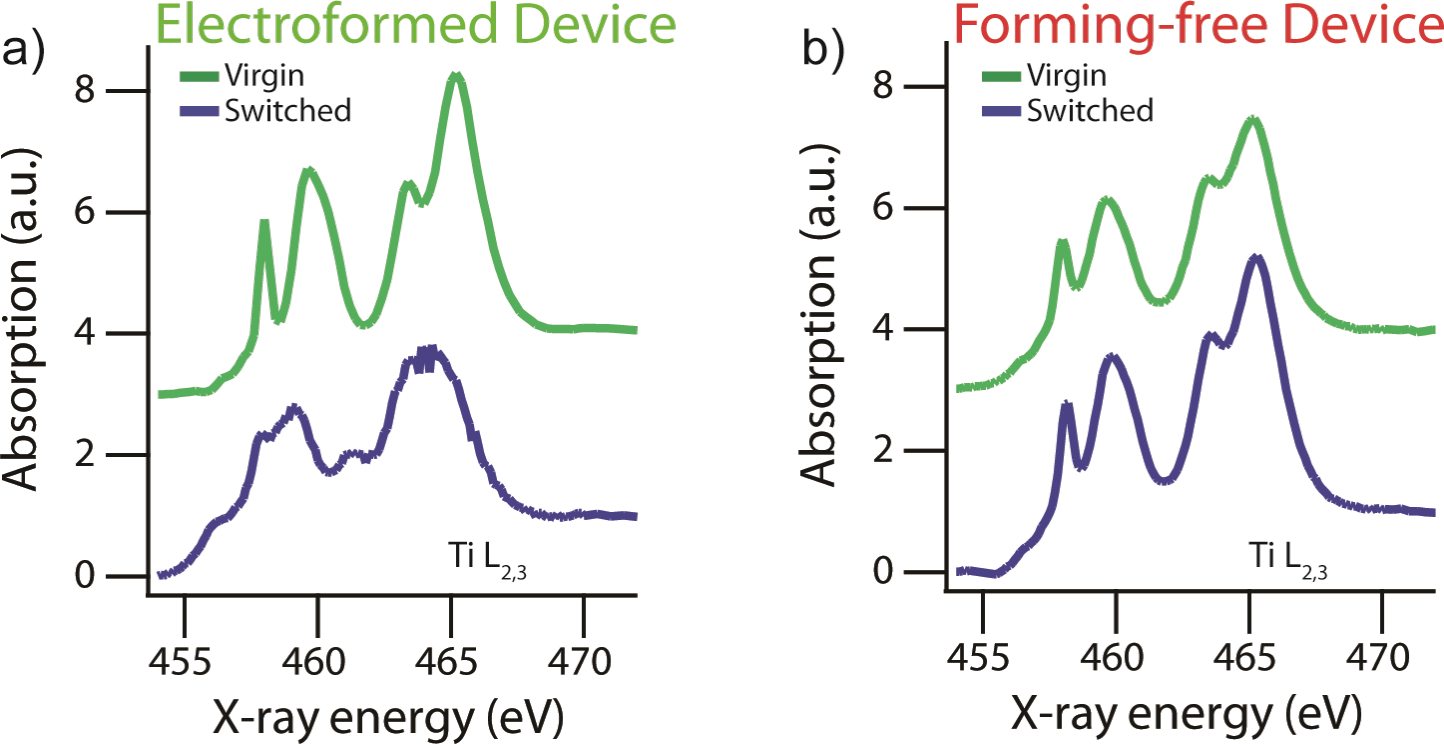
X-ray absorption spectroscopy within the junction region of an electroformed and forming-free device. The Ti L_3_ absorption edge is shown, which is sensitive to chemical composition and structure state. (a) The “Virgin” NEXAFS spectrum (green curve) was derived from a neighboring, simultaneously grown device which was not electrically biased, while the “switched” spectrum (blue curve) came from within the materially altered nanoscale region in the electroformed device of Figure 2 with a size of roughly 200 nm × 200 nm. (b) Both “Virgin” and “switched” spectra (green and blue curves, respectively) were derived from the junction region in the forming-free device of Figure 2, before and after the application of electrical bias, respectively.

X-ray absorption spectroscopy was also performed for both types of devices. In this work the NEXAFS of the Ti L_2,3_ edges of the film were integrated within the junction area. Figure 3 shows the spectra for electroformed (a) and forming-free (b) devices, in both cases comparing the Virgin material state to that after electrical switching. Within an electroformed device (Figure 3a) dramatic material changes were observed even when only comparing the switched and Virgin state qualitatively. The spectrum of the Virgin state showed four main peaks which are the L_3_ and L_2_ absorption peaks, each crystal field split into doublets (457–462 eV and 462–468 eV). The Virgin state spectrum is consistent with an amorphous state of the TiO_2_ [26,35]. In contrast, within the switched device, a NEXAFS spectrum was observed with a reduced crystal field splitting as well as a prominent lower energy absorption at 456 eV, assigned to the Ti^3+^ valence state. The NEXAFS therefore indicates a mixed-valence composition including both Ti^3+^ and Ti^4+^, and matches well the spectrum observed in reduced titanium oxide layers [36–38]. It is additionally worth noting that the spectrum observed in the ON and OFF state of the device was similar [26], suggesting that the material changes occurred during electroforming rather than during switching and that any material differences between the ON and OFF states were undetectable with this technique.

For the forming-free device containing a TiO_2−_*_x_* layer, the non-stoichiometry is qualitatively evident in the NEXAFS of the material grown, seen in the Virgin state (Figure 3b, green curve). Compared to the as-grown stoichiometric TiO_2_ layer (Figure 3a, green curve), this spectrum exhibits more merged and less prominent crystal field split doublets (457–462 eV and 462–468 eV). While not as distinct as the heavily reduced phase found in the electroformed layer (Figure 3a, blue curve), the trend is the same, showing that indeed the as-grown film for forming-free devices contains a substantial concentration of reduced valence titanium atoms and therefore oxygen vacancies.

Between the Virgin and switched states of the forming-free device, presented in Figure 3b, no large qualitative differences in the NEXAFS spectrum are observed. However, a very slight sharpening and increased absorption is seen in the spectrum after switching, which is likely caused by Joule heating from the applied current, which serves to anneal the film during operation [29,35] and to reduce disorder. The overall lack of material changes in the forming-free compared to the electroformed devices shows a progress in reducing the device damage during operation and can thus improve the technological promise of memristors. However, the presence of conductive channels less than 50 nm in areal diameter is not ruled out in this X-ray study, as spectroscopy of such small channels is at the limit of the present technique, and would require higher resolution electron-based measurements.

### Device characterization by TEM

**Figure 4:**
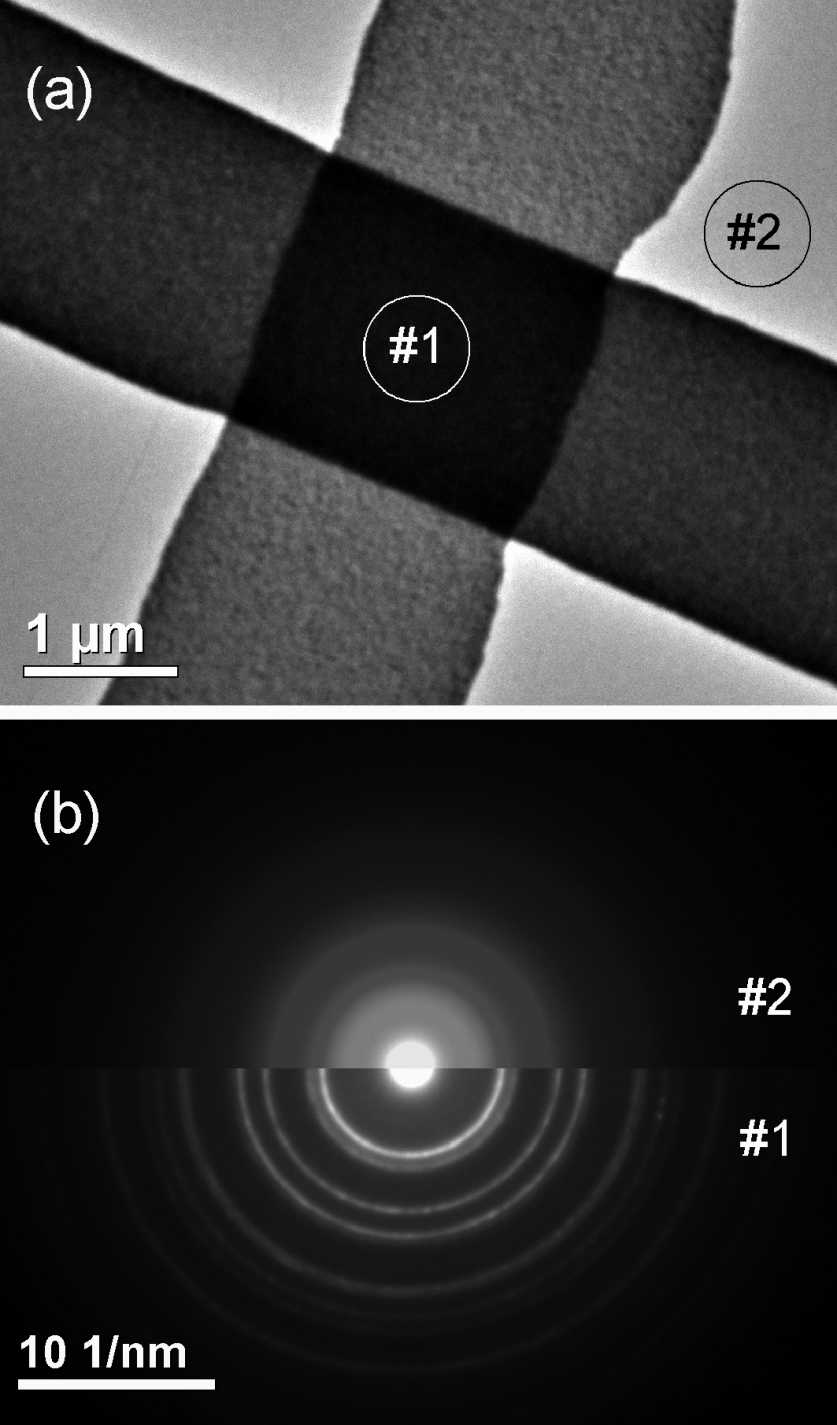
(a) Low-magnification TEM image of the junction region from a forming-free device in the ON state. (b) SAED patterns obtained in the two regions indicated in (a).

TEM characterization was performed using a JEM 2100F microscope. A customized single-tilt sample-holder tip was designed to accommodate the silicon/silicon-nitride substrates with the memristor device. The electron microscope was used to obtain selected area electron diffraction (SAED) patterns as well as for transmission and scanning-transmission imaging from bright-field and high-angle annular dark-field (HAADF) detection. Figure 4a shows a low-magnification TEM image of the post-switched forming-free device. A careful analysis of the junction region in the ON state indicates no clear evidence of local modifications as a result of the switching process. This fact was also suggested from a comparison among different images obtained from devices in ON, OFF and ‘Virgin’ states. Additionally, Figure 4b shows SAED patterns obtained through the regions indicated as #1 and #2 in Figure 4a. The diffraction patterns are dominated by the metallic elements and indicate polycrystalline material. The SAED pattern from region #1 (Pt/TiO_2−_*_x_*/TiO_2_/Pt) is dominated by the Pt electrodes. Region #2 (TiO_2−_*_x_*/TiO_2_ on silicon nitride) evidences only diffuse rings indicating predominantly amorphous material. Using different selecting apertures, other areas within the junction region were analyzed with equivalent results. Hence, these results indicate that the titanium oxide phases are amorphous, with no observation of structural or physical modifications in the junction originating from the switching process.

**Figure 5:**
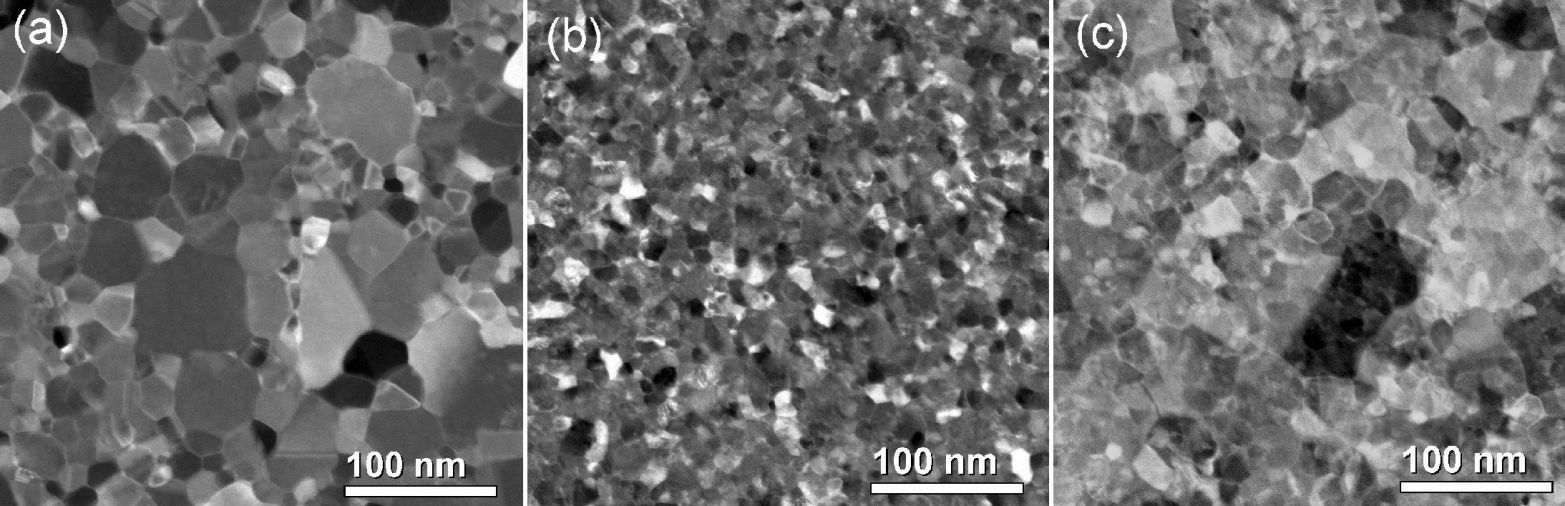
Bright-field STEM images obtained within different regions of the forming-free device: (a) top electrode, (b) bottom electrode, and (c) junction.

Scanning transmission electron microscopy (STEM) was also used to explore the forming-free device from a structural and morphological standpoint. Figure 5 depicts STEM images obtained from bright-field (BF) detection. BF imaging was utilized rather than HAADF detection due to the latter having an image contrast mainly dominated by the local atomic mass. In addition, the BF imaging has a major electron-phase contribution and channeling effect, which are more appropriate for probing the switching process. Nonetheless, no evidence of a switching effect was observed from a careful analysis along the junction. However, BF image analysis indicated that the Pt grains from the top electrode are larger than those of the bottom electrode, as seen in Figure 5a and Figure 5b. These grain size distributions were observed to be uniform within the junction, roughly 20 μm away at the Si_3_N_4_ window edge, and over the Si substrate outside of the window (as checked by scanning electron microscopy). This strongly suggests that the grain growth was initiated by self-heating in the thin film electrode itself.

## Conclusion

We have seen that it is possible to reduce and nearly eliminate many of the damaging effects which had been observed in titanium oxide based resistance switching devices [24–27] by engineering devices with an oxygen deficient layer which can serve as a vacancy reservoir. It was shown that, electrically, such a structure removed the need for a high voltage electroforming step and the as-fabricated device resistance closely matched the later attained OFF state. In addition, using several physical characterization methods with chemical and structural sensitivity and nanoscale spatial resolution, we saw that the cycling of the device did not bring about observable material changes. Thus, any such changes must be more subtle. One limitation of both techniques employed here is the lack of sensitivity in the direction perpendicular to the sample plane as these methods integrate through the entire layer stack in a transmission geometry. Thus, it is worthy of future study to explore the materials modifications in the perpendicular direction. Additionally, while the work presented here has shown the material state before and after electrical switching and the forming is performed ex-situ, in-situ studies, particularly with TEM, may be the best method to further probe and discover any minute material changes in a manner that can be directly correlated with the resistance switching in real time.
